# The effect of emotion regulation training on anxiety in college students in Egypt (Randomized control trial)

**DOI:** 10.1177/22799036251347030

**Published:** 2025-06-21

**Authors:** Amani Safwat ElBarazi

**Affiliations:** 1 Counseling and Mental Health Department, College of Education and Arts, Lusail University, Doha, Qatar

**Keywords:** COVID-19 pandemic, anxiety, emotion regulation, university students

## Abstract

**Background:**

The important role of Emotion Regulation in managing stress and anxiety is well recognized.

**Aims:**

(1) assessing the level of anxiety, and the level of difficulties in emotion regulation among University Students during Coronavirus disease 2019 (COVID-19), (2) investigating the effect of the emotional regulation training program on the anxiety of University Students during COVID-19.

**Methods:**

In part, I, 863 students at the British university in Egypt were assessed for their anxiety, difficulties in emotion regulation, and the general impact of COVID-19 on their lives. The students completed Beck Anxiety Inventory and Difficulties in Emotion Regulation Scale. Part II was a Randomized Control Trial using a pre-assessment and a post-assessment. A voluntary sample of 200 students was randomly assigned to either a group that received emotion regulation training or a control group. The training program is an adapted version of Dialectical Behavior Therapy skills training. The training program includes mindfulness, emotion regulation, and problem-solving skills through eight 90-min group sessions.

**Results:**

In Part I: The mean Beck Anxiety Inventory’s total score among students was 22.27 (SD = 13.6). Severe anxiety was reported by 39.9% of the students, and the mean difficulties in emotion regulation scale’s total score was 75.1 (SD = 23.24). In Part II, after the intervention, students in the experimental group, in comparison to the control group, resulted in significantly greater reductions in anxiety (the effect size, *d* = 0.53, *p* < 0.001) and significantly greater reductions in difficulties in emotion regulation (the effect size, *d* = 0.85, *p* < 0.001).

**Conclusion:**

The study findings indicate that training in emotional regulation considerably lowered students’ anxiety. It is recommended to teach the university students emotions regulation skills to improve their academic performance, enhance general well-being, and lower the risk of mental health concerns.

## Introduction

Coronavirus disease 2019 (COVID-19) has been recognized as a highly contagious respiratory disease that can be spread through droplets.^1^ On March 11, 2020, the World Health Organization declared a pandemic to reduce the spread of COVID-19. All educational institutes have remained closed since and shifted to alternative online learning. Such a shift leads to a profound impact on students’ daily activities and mental health.^2,3^ Quarantine is defined as the process of separating and restricting the movement of people exposed to a contagious disease to evaluate their health status and to reduce the risk of spreading the infection.^4^ On the other hand, isolation is the separation of people who have been diagnosed with a contagious disease from healthy ones.^5^

In the known history of humanity, several disease outbreaks have caused generalized fear in the public and anxiety-related behavior.^6,7^ COVID-19 has brought so many uncertainties with the possibility of a destructive outcome. Others have already reported manifestations of distress, anxiety, depression, and insomnia in general populations.^8,9^ Several studies reported negative psychological effects of COVID-19 such as stress, anger, uncertainty, confusion, depression, and even post-traumatic stress symptoms.^10–12^ People were anxious about infection, inadequate supplies, inadequate information, financial loss, stigma, and their future.^13^

University students usually are more prone to anxiety than other people because of experiencing several stressors during their academic lives.^14,15^ Stressors such as exams, fear of failure, and family and social conflicts affect students negatively regarding their grades at the university and their entire quality of life.^16^ Additional factors have come up related to fear of illness because of COVID-19.^17^

Recent research has shown a significant association between Emotion Regulation (ER) and mental health.^18^ People with higher levels of ER achieve more academic, cognitive, social, and affective goals successfully than people with lower levels of ER.^19^ Thus, stress and anxiety lead to emotional dysregulation, resulting in negative impacts on psychological and physiological health.^20^

ER is defined as “the process by which individuals influence which emotions they have, when they have them, and how they experience and express their feelings. ER can be automatic or controlled, conscious or unconscious, and may have effects at one or more points in the emotion-producing process.”^21^ Also, ER is defined as “the extrinsic and intrinsic processes responsible for monitoring, evaluating, and modifying emotional reactions, especially their intensive and temporal features, to accomplish one’s goal.”^22^ There are six components of ER: (1) emotional awareness, or the ability of an individual to understand his own emotions and of others; (2) emotional acceptance (3) emotional clarity (4) impulse control: the capacity to regulate one’s actions during times of emotional stress; (5) goals, or the ability to participate in goal-directed action while experiencing difficult emotions; and (6) strategies, or the capacity to use appropriate emotion management methods when stressed. ER functions as a modification to the information, it enables us to deal with stressors effectively and to respond adaptively in demanding and stressful situations.^23^

There are several approaches and therapies available for decreasing anxiety such as mindfulness, ER, assertiveness training and progressive muscle relaxation exercise.^24–27^ Some studies have been conducted to determine the effectiveness of emotion control training in decreasing stress and anxiety. For example, a controlled trial was conducted on university students to assess the effect of mindfulness meditation on anxiety levels and observed a substantial reduction in anxiety following seven consecutive 30-min daily mindfulness meditation meetings.^28^ Another randomized clinical study found that a 6-week daily meditation program combined with daily physical activity decreased depression, anxiety, and stress, as well as enhanced university students’ well-being and quality of life.^29^ Similarly, researchers investigated the benefits of a meditation training program on depression and anxiety in university students. Their findings revealed that meditation reduced students’ sadness and anxiety.^30^ Similarly, studies have shown the beneficial impact of progressive muscle relaxation exercise on nursing students’ anxiety levels in pediatric clinical settings.^25,31^ Correspondingly, a 3-month Dialectical Behavior Therapy Skills Training stand-alone outpatient treatment (DBT-ST-OP) was effective in reducing ER difficulties in individuals with alcohol use disorder (AUD).^32,33^

A meta-analysis sought to ascertain the relationship between enhanced emotional regulation and the outcomes of psychological therapy for depression and/or anxiety in adolescents. Improvements in depressed and anxious symptoms were positively correlated with enhancements in engagement and emotion regulation abilities.^34^

Hence, university students have been significantly affected by the psychological consequences of the COVID-19 pandemic, with anxiety emerging as one of the most prevalent mental health concerns during this period. Research indicates that difficulties in emotion regulation (ER) are strongly associated with increased vulnerability to anxiety, particularly in high-stress contexts such as global health crises. Emotion regulation training (ERT) is a structured psychological intervention that aims to enhance individuals’ capacity to understand, manage, and respond adaptively to emotional experiences. While ERT has demonstrated efficacy in reducing anxiety symptoms in various populations, there is a notable gap in research regarding its application among university students in the Middle East and North Africa (MENA) region, and more specifically, in Egypt. To address this gap, the current study was conducted with two primary aims: (1) assessing the levels of anxiety and difficulties in emotion regulation (DER) among university students during the COVID-19 pandemic, and (2) investigating the effect of an ERT program on anxiety levels in this population. It was hypothesized that students who received the ERT intervention would exhibit significantly reduced anxiety levels compared to those in the control group. Furthermore, it was predicted that improvements in emotion regulation would play a mediating role in this reduction. To the best of our knowledge, this is the first randomized controlled trial to examine the effectiveness of emotion regulation training on the anxiety of university students in Egypt during the COVID-19 pandemic.

## Methods

### Study design

This study consists of two parts, the first is a descriptive cross-sectional study, and the second is a two-parallel group, repeated-measure, randomized control trial design.

### Time and place of the study

The study was conducted between April 2021 and May 2022. The study was completed on the 1st of May 2022. The study was carried out at the British University in Egypt. First student enrollment began on April 2, 2021

### Population and sample

All individuals were university students and met the inclusion criteria. The inclusion criterion was as follows: Being an active student at a private university in Egypt. Exclusion criteria were as follows: (1) Receiving treatment at the hospital during the time that the study was in progress, (2) Having been diagnosed with psychotic disorders, and (3) Having cognitive difficulties. We confirm that written informed consent was obtained from all participants prior to participation, in accordance with COPE guidelines.

### Randomization and masking

Web-based randomization software (http://randomizer.at) has been used to allocate students to experimental or control groups in a 1:1 ratio. Due to the nature of the psychological intervention, neither participants nor facilitators were blinded to group assignments. Outcome assessment and data analysis were also conducted without blinding, which may introduce potential bias and is acknowledged as a limitation.

### Data collection tools

Standardized e-questionnaires were generated using Microsoft Forms, and the links were shared through students’ university emails and social media—Facebook. Also, the study data were collected using:

#### Personal information and questions related to the COVID-19 situation

include closed-ended questions about participants’ sociodemographic characteristics, and personal, family-related, social, financial, educational, and academic-related variables. There were questions related to the COVID-19 pandemic. Students were asked about Health risks and behavior-related variables such as smoking cigarettes, binge eating, cannabis, and alcohol consumption. Students were asked how many times they had five or more binges, alcoholic drinks, drugs, marijuana/hash, or any substance on one occasion during the past 30 days.

#### Beck anxiety inventory (BAI)

BAI Was developed by Beck et al., and it is a brief measure of anxiety with a focus on somatic symptoms of anxiety such as nervousness, dizziness, and inability to relax.^35^ It has a total of 21 items that take approximately 10–15 min to complete. Answers are on a 4-point Likert scale and range from 0 (not at all) to 3 (severely). The values for each item are added together to get an overall or total score for all 21 symptoms that can vary from 0 to 63 points. A total score of 0–21 indicates “Low” anxiety; 22–35 indicates “Moderate” anxiety; and 36–63 indicates “Severe” anxiety. The BAI also displays strong concurrent validity, with correlations with the SCL-90 Anxiety Subscale ranging between 0.78 and 0.81.^36,37^ The reliability of BAI for the current study: Internal consistency for the BAI=(Cronbach’s α= 0.92). Test-retest reliability (1 week) for the BAI was =0.75. The Beck Anxiety Inventory was standardized and validated for the Egyptian population.^38^

#### Emotion regulation difficulties

DERS is a 36-item self-reported questionnaire that assesses an individual’s ability to regulate their emotions.^23^ Participants rate items on a 5-point Likert scale (1 = seldom–5 = nearly frequently) to represent their self-perceived reactions to stressful emotional situations. The DERS produces an overall difficulty score in ER as well as six subscales: (a) Awareness, (b) Clarity, (c) Nonacceptance, (d) Impulsivity, (e) Goals, and (f) Strategies. Subscales are evaluated in such a way that higher values imply more difficulty. DERS has strong evidence supporting its validity among college students.^39^ The DERS subscales exhibited good internal consistency in the current study with Cronbach’s α values as follows: (a) Awareness (α = 0.84), (b) Clarity (α = 0.82), (c) Nonacceptance (α = 0.89), (d) Impulsivity (α = 0.94), (e) Goals (α = 0.83), and (f) Strategies (α = 0.82). DERS was standardized and validated for the Arabic population.^40^

### Study process

Part I includes a total of 863 university students from a private university in Egypt who were asked to complete the personal information questionnaire and questions related to COVID-19, BAI, and DERS. The students were asked to complete the questionnaires through Microsoft Forms over the internet as online questionnaires. The students needed approximately 25–35 min to answer the questionnaires. We asked each student during Part I if he/she would like to participate in the experiment in Part II. In part II, 200 students agreed to participate in the experiment and were randomized to either the experimental or control group. In both experimental and control groups, assessments of anxiety and DER were collected from all students two times, 3 months apart. Students in the experimental group (Emotion Regulation group) had an emotion regulation training program and met weekly for 10 weeks. There were seven experimental groups, each group comprising 14 students except for one group that had 16 students. Furthermore, students in the ER group were allowed to conduct telephone consultations and email communications to help them with their homework and exercises related to the training (see CONSORT 2025 Flow Diagram in Appendix A). The reporting of this study conforms to the CONSORT 2025 Statement for randomized controlled trials^41^ (Supplementary File).”

### Ethics and human subjects issues

All Students’ data and demographic information are stored at OneDrive of the British University in Egypt. Approval from the British University in Egypt Institutional Review Board was obtained to conduct the study (IRB Protocol CL-2008). The study was registered in Clinical Trials (ClinicalTrials.gov Identifier: NCT04932369) with all details about the two arms of the underlying investigation. Students signed up for the information sheet and consent form. They were informed of the nature & procedure, the aims of the study, the confidentiality of data, the choice of participating in the study, and the right to withdraw at any time from the study. Also, participants were monitored for any adverse psychological reactions during and after each session. Any reports of distress were documented by the facilitator and followed up with appropriate support. Also, harms were defined as any undesirable psychological or physical experiences that occurred during or shortly after participation in the intervention sessions, including heightened anxiety, emotional distress, or withdrawal symptoms. Harms were assessed systematically at the end of each session using a brief structured checklist administered by the facilitator, and participants were encouraged to report any discomfort experienced during the intervention period. Any reported harm was documented and reviewed by the research team.

*Interim Analyses and Stopping Guidelines*: No interim analyses were conducted, and no formal stopping guidelines were established for this study, as the intervention was considered low-risk, and the sample size was predetermined.

**Study therapists:** There was one clinical psychologist (Dr. ElBarazi) who was trained to administer DBT. Each group meeting was held by a clinical psychologist once a week (meeting 90 min).

### Training program

The training sessions were based on the Dialectical Behavior Therapy training manual.^42^ Handouts and worksheets inside the sessions were taken from Dialectical Behavior Therapy skills training handouts and worksheets.^43^ students were asked to practice and do exercises that were taken from the Dialectical Behavior Therapy Skills Workbook’.^44^ Each training group received one pre-training session to introduce the training program and eight 90-min sessions of emotion regulation training (one session per week). The training includes group gathering, discussion, and listening to students’ experiences. Homework and exercises between sessions were sent to the students by email. Also, telephone consultations were provided as needed. The training content was shown in Table 1.

**Table 1.** DBT content per session.

Session no	Main topic	Content
Pre-session	Introduction	A brief overview of the emotion regulation training topics will be addressed in each session. The main concepts and aims of emotion regulation were explained.
1	Understand and name Emotions	Students were taught skills such as being aware of emotions and naming and describing emotions. Identifying the primary & secondary emotions and their functions. Explaining the factors that prevent regulating strong emotions.
2	Mindfulness	Educating mindfulness skills is important in regulating emotions. Being mindful of emotions without judgment. Teaching the participants, the relaxation exercises, and methods of overcoming the barriers to healthy emotions. Introducing strategies for living in the present moment.
3	Acceptance	Educating the acceptance techniques (without judgment or evaluation) of both positive and negative emotions.
4	Decrease the frequency of unwanted emotions	Recognizing: emotions, behaviors, thoughts, and how these parts of personality interact with each other. Teach the participants ways to be fully aware of them. Describing techniques for preventing undesirable emotions from developing in the first place. Explaining techniques for changing undesirable feelings after they have begun.
5	Decrease emotional vulnerability	Increasing resilience refers to the capacity to deal with both tough and good emotions in effective ways. Reducing emotional vulnerability as well as physical and cognitive vulnerability.
6	Decrease emotional suffering	Reducing suffering when participants are overwhelmed by distressing emotions. Managing strong emotions so that they don’t aggravate the situation.
7	Emotion regulation Skills 1	Check the Facts: Determine whether emotional reactions are consistent with the facts of the issue. Changing or modifying thoughts and assumptions to match the facts might aid in altering emotional reactions to situations. Emotional exposure and performing the opposite of one’s emotional desires when emotions do not match the facts, or when acting on a person’s feelings is ineffective. Acting the opposite of one’s emotional urges will modify the emotional reactions in the situations.
8	Emotion regulation skills 2 (Problem-solving)	When the facts are the issue, resolving the issue will minimize the occurrence of unpleasant feelings. Teaching the participant to identify the ABCs of problem-solving in which (A. Alternatives. Brainstorm alternative responses. B. Best ideas C. Commitment to implementation). Students were taught to evaluate the list of choices and choose the best ideas to implement and identify the time and place to try the new responses.
Ending	Revision of the techniques	Revising the methods to cope with the situations in healthy ways Answering queries from students, concluding, and ending.

### Supervision and fidelity

The audio of the sessions was recorded with the agreement of all participants to assess adherence to the DBT principles. The sessions were split into modules of 15 min, which were selected at random for compliance checks. A DBT researcher and psychotherapist who was not participating in the current training program later reviewed the session content. Based on the Dialectical Behavior Therapy manual,^42^ the training program’s procedures were evaluated, and the modules were assessed as acceptable or not. It was considered that most of the sessions (89%) were done properly.

### Treatment adherence

All the students in the experimental and control groups came to participate in assessments 1 and 2 since their involvement was considered an extra activity. Their involvement was rewarded in the form of extra credit for their undergraduate psychology courses. All students in the Er group attended at least seven training sessions.

### Outcome measures

The primary outcome measures were BAI and DERS. All primary outcomes were collected at baseline and 3 months. SPSS Sample Power was to ensure that the sample size was sufficient to detect meaningful differences in primary outcomes. We set the following parameters based on previous research: the two-tailed test of significance, desired power = 0.80, unstructured covariance matrix, two-time points, correlation = 0.50 between repeated assessments, and Margin of error = 5%. With (200 participants), the study has 78% power to detect a medium effect size of 0.65 for group differences in outcomes.

### Data analysis

The data were evaluated using the IBM SPSS Statistics, version 23 software program. Descriptive statistics (means, standard deviations, frequencies, percentages) were used to describe the sociodemographic and baseline characteristics of this sample. Anxiety and DERS outcomes were modeled using generalized estimating equations (GEE).^45^ To model individuals across time points, a temporal within-subjects autoregressive [AR] correlation matrix was utilized. The distributions of the outcome measures were used to specify the models. BAI and DERS scores were modeled using identity link functions for normal distributions. Bivariate analyses were utilized as a part of the primary omnibus analysis to compare demographics and baseline symptom severity between the ER group and the control group. All analyses were conducted on the sample that was intended to be treated. We used GEE because it extends the generalized linear model, which processes corresponding data from repeat measurements, requires no parametric distribution assumption and robust inference for an incorrect description of the internal correlation of subjects, and provides good indications of within-subject correlations.^46^ At the α = 0.05 threshold, both simple and main effects were judged significant (two-tailed).^47^ To account for Type I errors, Bonferroni corrections were used for all models of BAI and DERS outcomes. Missing data were handled using complete case analysis, whereby only participants with complete pre- and post-intervention data were included in the final analysis. The number and proportion of participants lost to follow-up were reported, and reasons for missing data were documented where available. No subgroup or sensitivity analyses were conducted. All analyses were based on the primary and secondary outcomes as prespecified in the study protocol.

## Results

### Part I

There were (*n* = 863) university students who completed BAI and DERS. There was a significant positive correlation (*r* = 0.76, *p* < 0.000) between students’ difficulties in emotion regulation and their anxiety.

#### Demographic characteristics, BAI, and DERS scores

Table 2 presents baseline demographic variables, descriptive characteristics of the sample, and their correlation with BAI & DERS scores. The mean BAI total score among students was 22.27 (SD = 13.6). Severe anxiety was reported by 39.9% of the students, moderate anxiety was reported by 24.6% of the students, and low anxiety was reported by 17.3% of the students. No differences were found between the demographic characteristics of the students and their BAI scores except for loneliness, in which students who live with their families had higher anxiety scores on BAI compared to students who live alone (*M* difference = −21, *CI95*: −22.4 to −19.6, *p* < 0.000).

**Table 2.** Comparison of participants’ sociodemographic characteristics, anxiety, and difficulties in emotion regulation scores.

Variables		*n*	%	BAI			DERS		
		863	100	Mean (SD)	*t* or *F*	Sig	Mean (SD)	*t* or *F*	Sig
Gender	Males	283	32.8	22.13 (13.6)	0.03	0.84	74.5 (22.6)	0.25	0.61
	Females	580	67.2	22.33 (13.6)			75.3 (23.5)		
Marital status	Single	863	100	22.27 (13.6)	-	-	75.1 (23.24)	-	-
	Married	0	0	-			-		
University’s year	Year 1	371	43	22.17 (13.3)	0.033	0.85	76.02 (21.2)	1.04	0.30
	Year 2	492	57	22.34 (13.9)			74.3 (24.6)		
Region of residence	Urban	863	100	22.27 (13.6)	-	-		-	-
	Rural	0	0	-					
Income status	Income lower than expenses	258	29.9	21.8 (13.7)	0.45	0.63	73.1 (24.1)	1.4	0.23
	Equal income and expenses	456	52.8	22.6 (13.8)			76.1 (23.4)		
	Income higher than expenses	149	17.3	21.7 (12.6)			75.2 (20.8)		
Loneliness	Alone	261	30.2	7.62 (3.75)	862.9	0.000	55.48 (15.3)	385.01	0.000
	Living with family	602	69.8	28.6 (11.2)			83.5 (20.8)		

The mean difficulties in emotion regulation as shown by DERS total score among students was 75.1 (SD = 23.24). The mean of students’ difficulties in emotional awareness was 10.1 (SD = 4.3), difficulties in emotional acceptance were 10.7 (SD = 4.5), difficulties in emotional clarity was 10.1 (SD = 4.5), their impulsivity was 16.1 (SD = 7.3), their difficulties in engaging in goal-directed behavior during the emotional distress was 14.1 (SD = 7.3), and difficulties in practicing the healthy emotion regulation strategies when emotionally distressed was 13.8 (SD = 6.2).

No differences were found between the demographic characteristics of the students and their DERS scores except for loneliness, in which students who live with their families had higher difficulties in emotion regulation compared to students who live alone (*M* difference = −28.11, *CI95*: −30.9 to −25.3, *p* < 0.000).

#### COVID-19 variables, BAI, and DERS scores

Table 3 provides the association between the COVID-19–related variables, BAI, and DERS scores. Anxiety and difficulties in emotion regulation scores were significantly (Ps < 0.000) higher among participants who reported having chronic illnesses, took a test after suspicion of COVID-19, had one at least of their family members took a test of suspicion of COVID-19, stated that they need psychological support, reported that their daily routines have changed, exposed to social media for long hours, reported being socially inactive, were scared of death, were smokers, experienced binge eating, experienced binge alcohol consumed hash, engaged in physical exercises, having effective coping skills and believed in conspiracy theories regarding the causes of COVID-19.

**Table 3.** Associations between factors related to COVID-19, BAI and DERS.

Variables		*n*	*%*	BAI			DERS		
		863	100	Mean (SD)	*t* or *F*	Sig	Mean (SD)	*t* or *F*	Sig
Do you have a chronic illness?	Yes	26	3.01	53.7 (2.9)	170.6	0.000	73.9 (22.4)	76.5	0.000
	No	837	96.9	21.2 (12.6)			112.7 (15.4)		
Did you take a test after you had a suspicion of COVID-19?	Yes	68	7.8	32.8 (19.3)	47.06	0.000	90.2 (25.6)	32.7	0.000
	No	795	92.1	21.3 (12.6)			73.7 (22.5)		
Did a family member of yours take a test after they had a suspicion of COVID-19?	Yes	150	17.4	42.2 (10.1)	714.4	0.000	69.9 (20.5)	258.9	0.000
	No	713	82.6	18.05 (10.07)			99.4 (19.6)		
Do you think you need psychological support during this period?	Yes	857	99.3	22.4 (13.5)	13.5	0.000	42.3 (19.2)	12.15	0.001
	No	6	0.7	2 (0.000)			75.3 (23.1)		
Effects of COVID-19	I was not affected at all	5	0.5	2 (0.000)	11.23	0.001	45.8 (19.3)	8.05	0.005
	My daily routines (such as meals, sleep, shopping) have changed	858	99.4	22.38 (13.5)			75.2 (23.1)		
Social media	No	636	73.6	18.18 (10.1)	288.6	0.000	70.33 (20.8)	114.9	0.000
	I was exposed to social media more than before	227	26.3	33.7 (15.6)			88.4 (24.5)		
Social life during COVID19	I was not affected at all	64	7.4	3.04 (0.86)	163.1	0.000	51.4 (14.7)	78.1	0.000
	Meetings with my friends, relatives, and neighbors have become rare	799	92.6	23.8 (13)			76.9 (22.7)		
Fear of death	Yes	843	97.7	22.7 (13.3)	55.4	0.000	42.8 (14.9)	41.3	0.000
	No	20	2.3	0.50 (0.51)			75.8 (22.8)		
Smoking	Yes	99	11.5	46.6 (5.03)	607.2	0.000	104.5 (16.5)	227.1	0.000
	No	764	88.5	19.11 (11)			71.2 (21.1)		
Binge eating	Yes	121	14	35.7 (16.6)	164.3	0.000	92.2 (24.4)	84.4	0.000
	No	742	86	20.06 (11.7)			72.2 (21.8)		
Using alcohol	Yes	63	7.3	49.2 (4.4)	385.4	0.000	108.03 (15.2)	161.8	0.000
	No	800	92.7	20.1 (11.7)			72.5 (21.7)		
Using drugs or hash	Yes	39	4.5	51.9 (3.5)	249.5	0.000	109.3 (15.05)	98.4	0.000
	No	824	95.5	20.8 (12.2)			73.4 (22.3)		
Coping strategies (reading a book, watching movies, going on a walk-in open area, Exercising, etc.)	Yes	152	17.6	4.01 (2.07)	535.2	0.000	51.15 (15.6)	252.9	0.000
	No	711	82.4	26.1 (11.7)			80.2 (21.3)		
Conspiracy theories	Yes	545	63.2	30.5 (9.8)	1445.02	0.000	85.9 (19.5)	519.6	0.000
	No	318	36.8	8.14 (4.5)			56.4 (16.1)		

### Part II

#### The randomized control trial

The control group contained (*n* = 100) students, of which (*n* = 61) were female students, whereas the experimental group included (*n* = 100) students, of which (*n* = 55) were female students. The experimental group included (*n* = 70) year 1 students and (*n* = 30) year 2 students, while the control group included (*n* = 85) year 1 students and (*n* = 15) year 2 students.

Table 4 presents the demographic characteristics of experimental and control groups. No differences were discovered between the group’s characteristics except for the “academic year” variable.

**Table 4.** Comparison of participants’ sociodemographic characteristics for the experimental and control groups.

Variables	Subtypes of Variables	Control		Experimental		Total		χ*2*	Sig
		*n*	%	*n*	%	*n*	%		
Gender	Males	39	19.5	45	22.5	84	42	0.7	0.4
	Females	61	30.5	55	27.5	116	58		
University’s year	Year 1	85	42.5	70	35	155	77.5	6.4	0.009
	Year 2	15	7.5	30	15	45	22.5		
Income status	Income lower than expenses	18	9	32	16	50	25	5.2	0.07
	Equal income and expenses	56	28	46	23	102	51		
	Income higher than expenses	26	13	22	11	48	24		
Loneliness	Alone	40	20	33	16.5	73	36.5	1.05	0.3
	Living with family	60	30	67	33.5	127	63.5		

Table 5 presents the differences between the experimental and control groups regarding COVID-19 variables. No differences were identified between groups regarding COVID-19 factors.

**Table 5.** Associations between factors related to COVID-19 and the experimental and control groups.

Variables		Control		Experimental		*Total*		χ2	Sig
		*n*	%	*n*	%	*n*	%		
Do you have a chronic illness?	No	93	46.5	97	48.5	190	95	1.6	0.3
	Yes	7	3.5	3	1.5	3	10		
Did you take a test after you had a suspicion of COVID-19?	No	97	48.5	96	48	193	96.5	0.1	1
	Yes	3	1.5	4	2	7	3.5		
Did a family member of yours take a test after they had a suspicion of COVID-19?	No	77	38.5	77	38.5	154	77	0	1
	Yes	23	11.5	23	11.5	46	23		
Effects of COVID-19	I was not affected at all	5	2.5	4	2	9	4.5	0.1	1
	My daily routines (such as meals, sleep, shopping) have changed	95	47.5	96	48	191	95.5		
Social media	No	60	30	59	29.5	119	59.5	0.02	1
	I was exposed to social media more than before	40	20	41	20.5	81	40.5		
Social life during COVID19	I was not affected at all	10	5	8	4	18	9	0.2	0.8
	Meetings with my friends, relatives, and neighbors have become rare	90	45	92	46	182	91		
Fear of death	No	5	2.5	6	3	11	5.5	0.09	1
	Yes	95	47.5	94	47	189	94.5		
Smoking	No	82	41	88	44	170	85	1.4	0.3
	Yes	18	9	12	6	30	15		
Binge eating	No	82	41	85	42.5	167	83.5	0.3	0.7
	Yes	18	9	15	7.5	33	16.5		
Consuming alcohol	No	89	44.5	92	46	181	90.5	0.5	0.6
	Yes	11	5.5	8	4	19	9.5		
Using drugs or hash	No	93	46.5	95	47.5	188	94	0.3	0.7
	Yes	7	3.5	5	2.5	12	6		
Coping strategies (reading a book, watching movies, going on a walk-in open area, Exercising, etc.)	No	20	10	26	13	46	23	1.01	0.4
	Yes	80	40	74	37	154	77		
Conspiracy theories	No	26	13	40	20	66	33	4.1	0.06
	Yes	74	37	60	30	134	67		

Table 6 shows that the BAI and DERS scores of the ER and control groups did not differ before the intervention (*p* > 0.05).

**Table 6.** Observed means of BAI and DERS at baseline and end-of-treatment (3 months) with model-based treatment effects (for the experimental and control groups).

Outcomes	ER	Control	TOTAL	Estimate	95% CI		*p*
BAI total	M (SD)	M (SD)	M (SD)				
Baseline	19.9(8.5)	21.8(8.9)	20.9(8.7)	_	_	_	_
End-of-treatment	17.3(8.4)	21.9(8.8)	19.6(8.9)	−2.6	−2.9	−2.4	0.000
DERS total	M (SD)	M (SD)	M (SD)	Estimate	95% CI		*p*
Baseline	95.3(20.2)	93.7(23.6)	94.5(21.9)	_	_	_	_
End-of-treatment	76.6(17.3)	92.1(19.1)	84.3(19.7)	−17.1	−19.5	−14.6	0.000
Difficulties in emotional Awareness	M (SD)	M (SD)	M (SD)	Estimate	95% CI		*p*
Baseline	12.2(3.5)	12.05(4.04)	12.1(3.8)	_	_	_	_
End-of-treatment	9.3(3.8)	12.2(4.1)	10.7(4.2)	−3.1	−3.7	−2.3	0.000
Difficulties in emotional Clarity	M (SD)	M (SD)	M (SD)	*Estimate*	95% CI		*p*
Baseline	15.7(5.6)	13.5(3.03)	14.6(4.6)	-	-	-	-
End-of-treatment	13.8(4.8)	13.3(3.01)	13.6(4.1)	−1.6	−2.2	−1.07	0.000
Non-acceptance	M (SD)	M (SD)	M (SD)	Estimate	95% CI		*p*
Baseline	13.1(5.1)	12.6(4.1)	12.9(4.5)	-	-	-	-
End-of-treatment	10.9(5.2)	12.9(4.6)	11.9(5.03)	−2.5	−3.1	−1.8	0.000
Emotional Impulsivity	M (SD)	M (SD)	M (SD)	Estimate	95% CI		*p*
Baseline	19.8(6.2)	20.7(5.8)	20.3(6.04)	-	-	-	-
End-of-treatment	16.9(6.5)	19.5(5.6)	18.2(6.2)	−1.7	−2.8	−0.7	0.001
Difficulties in setting Goals	M (SD)	M (SD)	M (SD)	Estimate	95% CI		*p*
Baseline	16.7(6.6)	18.4(6.6)	17.5(6.6)	-	-	-	-
End-of-treatment	10.6(5.3)	18.1(6.2)	14.3(6.9)	−5.7	−6.8	−4.5	0.000
Difficulties in planning Strategies to achieve goals	M (SD)	M (SD)	M (SD)	Estimate	95% CI		*p*
Baseline	17.5(6.2)	16.3(6.5)	16.9(6.4)	-	-	-	-
End-of-treatment	14.8(5.7)	15.8(6)	15.3(5.8)	−2.2	−3.2	−1.3	0.000

*Note.* BAI: beck anxiety inventory; CI = confidence interval; DERS = difficulties in emotion regulation; ER: emotional regulation.

The mean BAI of the control group during the pretest was 21.8 (SD = 8.9), but in the posttest, it was 21.9 (SD = 8.8). The mean BAI of the ER group during the pretest was 19.9 (SD = 8.5), but at the posttest, it was 17.3 (SD = 8.4). At assessment 2, the emotion regulation group (ER), in comparison to the control group, resulted in significantly greater reductions in anxiety as shown in BAI scores (the effect size, *d* = 0.53, *p* < 0.000).

Table 6 shows that the mean DERS of the control group during the pretest was 93.7 (SD = 23.6), but in the posttest, it was 92.1 (SD = 19.1). The mean DERS of the ER group during the pretest was 95.3 (SD = 20.2), but at the posttest, it was 76.6 (SD = 17.3). At assessment 2, ER group, in comparison to the control group, resulted in significantly greater reductions in difficulties in Emotion regulation as shown in total DERS scores (the effect size, *d* = 0.85, *p* < 0.000).

At assessment 2, as shown in DERS’ subscale scores ER group in comparison to the control group resulted in significantly greater reductions in difficulties in emotional awareness (the effect size, *d* = 0.7 *p* < 0.000), significantly greater reductions in difficulties in emotional clarity (the effect size, *d* = 0.1 *p* < 0.000), significantly greater reduction in difficulties in acceptance (the effect size, *d* = 0.4 *p* < 0.000), greater reductions in impulsivity (the effect size, *d* = 0.4 *p* < 0.000), significantly greater reductions in difficulties in engaging in goal-directed behavior during the emotional distress (the effect size, *d* = 1.3 *p* < 0.000), and significantly greater reductions in difficulties in practicing the healthy emotion regulation strategies when emotionally distressed (the effect size, *d* = 0.1 *p* < 0.000).

#### Harm or adverse events

No serious adverse events were reported in either group. In the intervention group, 2 participants (2%) reported temporary emotional distress during early training sessions, which were resolved without intervention. In the control group, 3 participants (3%) reported a mild increase in anxiety.

## Discussion

The COVID-19 pandemic is a public health emergency that demands effective training programs throughout the healthcare system. University students are particularly more prone to concerns regarding mental health.

In part I of the study we investigated the prevalence of anxiety and difficulty in ER among university students, and the association between Anxiety, DER, and COVID-19 variables. The results showed moderate levels of anxiety and a mild level of difficulty in ER. Our results showed that among university students there was a significant positive association between difficulties in emotion regulation and anxiety. The more the student’s issues with emotion regulation, the greater his anxiety. Students scored differently on the DERS subscales in order from highest to lowest: impulsiveness, difficulties in goal-directed behavior during emotional distress, difficulties in practicing healthy emotion regulation strategies when emotionally distressed, difficulties in emotional acceptance, clarity, and awareness. Our findings are congruent with those who found that greater levels of emotion regulation difficulties were related with COVID-19 among college students during the pandemic.^48^

COVID-19 has negatively impacted a large portion of university students in which anxiety and difficulties in emotion regulation were significantly higher among students who lived with their families, who reported that they or at least one of their family members took a test after suspicion of COVID-19, their daily routines (such as meals, sleep, shopping) have changed, being socially inactive and rarely meet with their friends, relatives, and neighbors, being exposed to social media more than before COVID-19 lockdown, smokers, binge eater, binge alcohol, hash, not engaging in physical exercise, not having effective coping skills such as reading a book and believed in conspiracy theories regarding the causes of COVID-19. Our findings are consistent with those who found that greater levels of ER difficulties were related with COVID-19 lifestyle modifications among college students during the pandemic^49^

The ongoing COVID-19 pandemic is creating psychological difficulties such as increasing cleaning obsessions, anxiety, sleeping disorder, and depression among young populations.^50^ One reason could be that most people all over the world experience anxiety and fear of illness (COVID-19). Most people have an uncertain, unclear, and negative view of the future. Students during the lockdown may become pessimistic and decrease in their interests.^2^ They have become unsure of deciding anything important in their lives because of their worries about the future. Besides, quarantine reduced social activities which resulted in decreasing social and emotional support.^51^ Another possible reason for our results might be watching and reading COVID-19 news daily for longer hours than before the lockdown. Different studies have shown that watching and reading COVID-19 news excessively and for long hours can cause psychological difficulties such as anxiety, and depression.^52–54^

In part II of the study, we investigated the effect of ER training on anxiety in university students. Part II featured eight 90-min group sessions teaching students about ER, in addition to the precession and an ending session. The results showed that anxiety and difficulties in ER scores significantly decreased in the experimental group compared to the control group. We taught students emotion regulation skills and mindfulness techniques. In the emotional regulation group, students learn acceptance skills toward self and others, being nonjudgmental, being present, enjoying the present-moment experiences, and relaxing techniques. Students’ anxiety can be improved by reducing the difficulties in emotion regulation. Our results were in the same line with the meta-analysis’s results that claimed that difficulties in emotion regulation were associated with anxiety, depression, eating, and substance-related disorders among nonclinical and clinical samples.^55^ Also, previous studies showed the effectiveness of emotion regulation training programs in improving the anxiety of participants.^56^

Emotion regulation training (ERT) in the experimental group decreases anxiety through a variety of interconnected mechanisms that assist individuals in managing and altering their emotional reactions. These strategies may include: (1) Cognitive Reappraisal (altering one’s interpretation of circumstances). ERT frequently teaches cognitive reappraisal, in which people learn to reframe anxiety-provoking stimuli in a more neutral or positive way. Individuals can minimize the severity and frequency of anxious sensations by reframing events that cause them to arise.^57^ (2) Increasing Emotional Awareness, which assists people in recognizing early indicators of worry and distinguishing between different emotions. Being more emotionally aware enables people to intervene before anxiety worsens.^58^ (3) Mindfulness and present-moment focus enable students to be present and accept their feelings without judgment. This reduces ruminative thinking and worries about the future, which are major contributors to anxiety.^59^ (4) Reducing emotional avoidance: Anxiety frequently causes students to avoid events or feelings that they perceive as dangerous or threatening. ERT promotes students to confront their emotions rather than avoid them, which reduces their effects on the students.^60,61^ (5) Improving self-regulation and emotional control: Techniques like breathing exercises, grounding, and relaxation can help reduce physiological and emotional arousal in anxiety-provoking circumstances.^62,63^ (6) Shifting focus from threat to safety as anxiety is typically caused by an exaggerated perception of threat. Students can use ERT to recognize and redirect their attention away from perceived risks and toward safety signals or non-threatening parts of a scenario. Shifting Focus from Threat to Safety decreases hypervigilance and the “fight-or-flight” response, hence minimizing anxiety-related behaviors.^64^ (7) Improving Emotional Tolerance and Resilience by teaching students how to handle unpleasant emotions without being overwhelmed or prompted to respond impulsively. Over time, this improves emotional resilience. Improving emotional tolerance and resilience improves students’ ability to cope with stressful events, resulting in a reduction in anxiety over time.^65^ (8) Breaking negative thought patterns (cognitive difusion): ERT frequently includes tactics for teaching students to “defuse” from negative ideas. Anxious thoughts are not seen as absolute facts, but rather as fleeting mental experiences that may be recognized and let go of. This will reduce the ability of worried thoughts to trigger emotional reactions, limiting their influence on behavior and moods.^66^ (9) Promoting positive emotions through activities that encourage joy, gratitude or relaxation.^67^ Experiencing Positive emotions can help alleviate anxiety and promote emotional regulation. Promoting Positive Emotions improves psychological well-being and decreases the severity of negative emotional states such as anxiety.^68–70^

Students in both the experimental and control groups exhibited moderate difficulty managing their emotions during the COVID-19 situation. Our findings are consistent with the finding that unpleasant emotions might distort the meaning of events for students, leading to illogical behavior such as limiting home exercise because of COVID-19. This action was associated with negative feelings. They also observed emotional differences in the precautions students take, such as restricting outside activities or social meetings.^71^

Our findings suggest that the more the student’s issues with emotion regulation, the greater his anxiety levels were. Implementing interventions focused on improving emotional regulation could potentially help alleviate anxiety symptoms in this population. The unique contributions of our study highlight the connection between emotion regulation and anxiety levels in university students, as well as emphasizing the potential impact of targeted interventions in addressing these issues. By shedding light on this relationship, our study provides valuable insights for developing effective strategies to support student mental health and well-being.

The implications and recommendations of the present study can be to teach students skills that improve their emotion regulation to control their anxiety, fear, and stress. Also, they teach students mindfulness skills as students who manage their emotions effectively at the university usually have satisfying social relationships and less stress and anxiety. In addition, follow-up procedures to investigate how well the results were maintained might be carried out by the future study. Our research may include specific recommendations for applying emotion regulation training in educational contexts, such as introducing relaxing techniques into everyday routines and allowing students to practice emotion management skills in real-life circumstances. Furthermore, including parents and carers in the training process can assist in reinforcing these skills outside of the classroom and ensure consistency in supporting students’ emotional development.

The findings of this study are primarily applicable to undergraduate students in Egyptian university settings, as the sample was drawn from a single public university and consisted largely of young adults aged 18–26. While the inclusion of both male and female participants improves external validity, the results may not fully generalize to older adults, individuals with different educational backgrounds, or non-university populations. Moreover, the group-based, in-person delivery of emotion regulation training may not yield the same results if administered online or in individual sessions. Future studies should replicate this intervention in diverse cultural and educational settings to further assess its broader applicability.

Limitations and future directions: First, the participant’s ability and willingness to provide sensitive and personal information related to their fears, and difficulties in emotional experiences. Second, generalizing the results to people outside the university students should be done with caution. Third, future research may use longitudinal data on the association between anxiety and emotion regulation including a larger sample of participants. Fourth, the lack of information about the maintenance of the results because the follow-up measures were not taken. Also, the intervention was generally well-tolerated, with no serious adverse events reported. Temporary emotional distress in a small number of participants may reflect the emotionally evocative nature of regulation training and should be considered in future applications

As our results show that ERT reduces anxiety, the concrete steps for future research can be focused on exploring the specific mechanisms through which ER impacts anxiety levels. Additionally, examining the long-term effects of different ER strategies on overall mental health outcomes could provide valuable insights for developing more effective interventions.

## Conclusions

In conclusion, the results of this study focus on the impacts of pandemic-related disruptions on university students regarding their mental health and wellness. We found the prevalence of moderate anxiety and mild difficulties in ER among university students. Our results found a significant positive correlation between anxiety and difficulties in ER among university students. Our findings indicate that regulating emotions decreases anxiety in stressful settings, such as the COVID-19 pandemic. This shows that those who can effectively regulate their emotions may feel less anxious during times of crisis. The importance of our findings is critical for creating effective interventions for those suffering from anxiety disorders. This research highlights the importance of emotional regulation in promoting resilience and well-being in challenging circumstances. Although our results show that ERT reduces anxiety, additional research will be required to understand the mechanisms explaining how precisely regulating emotions reduces anxiety disorders.

## Supplemental Material

sj-docx-3-phj-10.1177_22799036251347030 – Supplemental material for The effect of emotion regulation training on anxiety in college students in Egypt (Randomized control trial) Supplemental material, sj-docx-3-phj-10.1177_22799036251347030 for The effect of emotion regulation training on anxiety in college students in Egypt (Randomized control trial) by Amani Safwat ElBarazi in Journal of Public Health Research

sj-pdf-1-phj-10.1177_22799036251347030 – Supplemental material for The effect of emotion regulation training on anxiety in college students in Egypt (Randomized control trial) Supplemental material, sj-pdf-1-phj-10.1177_22799036251347030 for The effect of emotion regulation training on anxiety in college students in Egypt (Randomized control trial) by Amani Safwat ElBarazi in Journal of Public Health Research

sj-pdf-2-phj-10.1177_22799036251347030 – Supplemental material for The effect of emotion regulation training on anxiety in college students in Egypt (Randomized control trial) Supplemental material, sj-pdf-2-phj-10.1177_22799036251347030 for The effect of emotion regulation training on anxiety in college students in Egypt (Randomized control trial) by Amani Safwat ElBarazi in Journal of Public Health Research

## Figures and Tables

**Figure:**
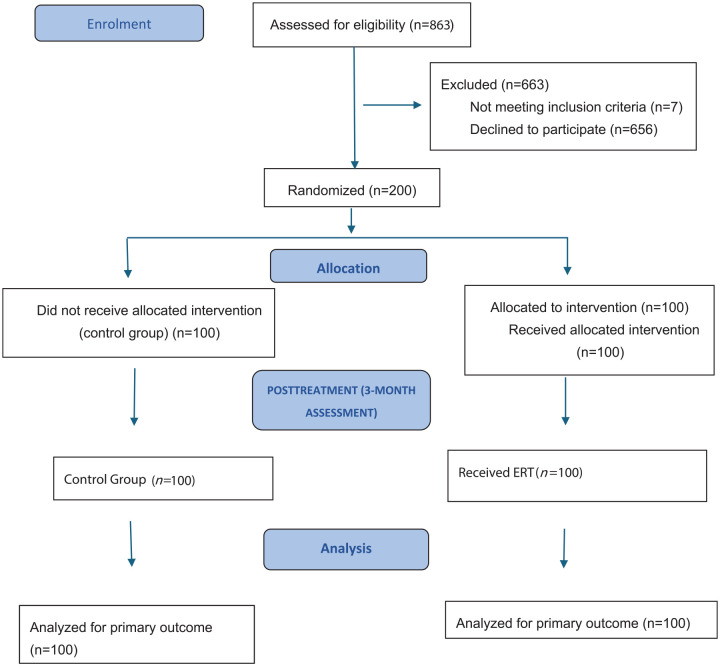
Flow diagram of the progress through the phases of a randomised trial of two groups.
