# Supplementary material for: The effect of emotion regulation training on anxiety in college students in Egypt (Randomized control trial)
Source: J Public Health Res. 2025 Jun 21;14(2):22799036251347030. doi: 10.1177/22799036251347030 (PMC12182610; doi:10.1177/22799036251347030)
Supplement: sj-pdf-2-phj-10.1177_22799036251347030 – Supplemental material for The effect of emotion regulation training on anxiety in college students in Egypt (Randomized control trial) [file sj-pdf-2-phj-10.1177_22799036251347030.pdf]

Difficulties in Emotion Regulation Scale (DERS)

Please indicate how often the following statements apply to you by writing the appropriate number from the scale below on the line beside each item.

1--------------------------2--------------------------3--------------------------4--------------------------5

almost never	sometimes	about half the time	most of the time	almost always
(0-10%)	(11-35%)	(36-65%)	(66-90%)	(91-100%)

_____ 1) I am clear about my feelings.
_____ 2) I pay attention to how I feel.
_____ 3) I experience my emotions as overwhelming and out of control.
_____ 4) I have no idea how I am feeling.
_____ 5) I have difficulty making sense out of my feelings.
_____ 6) I am attentive to my feelings.
_____ 7) I know exactly how I am feeling.
_____ 8) I care about what I am feeling.
_____ 9) I am confused about how I feel.
_____ 10) When I'm upset, I acknowledge my emotions.
_____ 11) When I'm upset, I become angry with myself for feeling that way.
_____ 12) When I'm upset, I become embarrassed for feeling that way.
_____ 13) When I'm upset, I have difficulty getting work done.
_____ 14) When I'm upset, I become out of control.
_____ 15) When I'm upset, I believe that I will remain that way for a long time.
_____ 16) When I'm upset, I believe that I will end up feeling very depressed.
_____ 17) When I'm upset, I believe that my feelings are valid and important.
_____ 18) When I'm upset, I have difficulty focusing on other things.
_____ 19) When I'm upset, I feel out of control.
_____ 20) When I'm upset, I can still get things done.
_____ 21) When I'm upset, I feel ashamed at myself for feeling that way.
_____ 22) When I'm upset, I know that I can find a way to eventually feel better.
_____ 23) When I'm upset, I feel like I am weak.
_____ 24) When I'm upset, I feel like I can remain in control of my behaviors.
_____ 25) When I'm upset, I feel guilty for feeling that way.
_____ 26) When I'm upset, I have difficulty concentrating.
_____ 27) When I'm upset, I have difficulty controlling my behaviors.
_____ 28) When I'm upset, I believe there is nothing I can do to make myself feel better.
_____ 29) When I'm upset, I become irritated at myself for feeling that way.
_____ 30) When I'm upset, I start to feel very bad about myself.
_____ 31) When I'm upset, I believe that wallowing in it is all I can do.
_____ 32) When I'm upset, I lose control over my behavior.
_____ 33) When I'm upset, I have difficulty thinking about anything else.
_____ 34) When I'm upset I take time to figure out what I'm really feeling.
_____ 35) When I'm upset, it takes me a long time to feel better.
_____ 36) When I'm upset, my emotions feel overwhelming.

Reverse-scored items (place a subtraction sign in front of them) are numbered 1, 2, 6, 7, 8, 10, 17, 20, 22, 24 and 34.

Calculate total score by adding everything up. Higher scores suggest greater problems with emotion regulation.

SUBSCALE SCORING:** The measure yields a total score (SUM) as well as scores on six sub-scales:

1. Nonacceptance of emotional responses (NONACCEPT): 11, 12, 21, 23, 25, 29
2. Difficulty engaging in Goal-directed behavior (GOALS): 13, 18, 20R, 26, 33
3. Impulse control difficulties (IMPULSE): 3, 14, 19, 24R, 27, 32
4. Lack of emotional awareness (AWARENESS): 2R, 6R, 8R, 10R, 17R, 34R
5. Limited access to emotion regulation strategies (STRATEGIES): 15, 16, 22R, 28, 30, 31, 35, 36
6. Lack of emotional clarity (CLARITY): 1R, 4, 5, 7R, 9

Total score: sum of all subscales

**"R" indicates reverse scored item

REFERENCE:

Gratz, K. L. & Roemer, L. (2004). Multidimensional assessment of emotion regulation and dysregulation: Development, factor structure, and initial validation of the Difficulties in Emotion Regulation Scale. *Journal of Psychopathology and Behavioral Assessment, 26,* 41-54.
